# Investigation of distributed learning for automated lesion detection in head MR images

**DOI:** 10.1007/s12194-024-00827-5

**Published:** 2024-07-24

**Authors:** Aiki Yamada, Shouhei Hanaoka, Tomomi Takenaga, Soichiro Miki, Takeharu Yoshikawa, Yukihiro Nomura

**Affiliations:** 1 Department of Medical Engineering, Graduate School of Science and Engineering, Chiba University, 1-33 Yayoi-Cho, Inage-Ku, Chiba, 263-8522 Japan; 2 Department of Radiology, The University of Tokyo Hospital, 7-3-1 Hongo, Bunkyo-Ku, Tokyo 113-8655 Japan; 3 Department of Computational Diagnostic Radiology and Preventive Medicine, The University of Tokyo Hospital, 7-3-1 Hongo, Bunkyo-Ku, Tokyo 113-8655 Japan; 4 Center for Frontier Medical Engineering, Chiba University, 1-33 Yayoi-Cho, Inage-Ku, Chiba, 263-8522 Japan

**Keywords:** Computer-aided detection (CADe), Distributed learning, Federated learning, Cyclical weight transfer, Cerebral aneurysm, Brain metastasis

## Abstract

In this study, we investigated the application of distributed learning, including federated learning and cyclical weight transfer, in the development of computer-aided detection (CADe) software for (1) cerebral aneurysm detection in magnetic resonance (MR) angiography images and (2) brain metastasis detection in brain contrast-enhanced MR images. We used datasets collected from various institutions, scanner vendors, and magnetic field strengths for each target CADe software. We compared the performance of multiple strategies, including a centralized strategy, in which software development is conducted at a development institution after collecting de-identified data from multiple institutions. Our results showed that the performance of CADe software trained through distributed learning was equal to or better than that trained through the centralized strategy. However, the distributed learning strategies that achieved the highest performance depend on the target CADe software. Hence, distributed learning can become one of the strategies for CADe software development using data collected from multiple institutions.

## Introduction

Numerous research groups have developed computer-aided detection (CADe) software, and recently, there has been an increase in CADe software using machine learning, including deep learning [1–6]. The performance of CADe software using machine learning depends on the quality and quantity of the data used for machine learning models. If the characteristics of data differ between development and clinical use, the expected performance of CADe software may not be achieved. The factors contributing to data heterogeneity include differences in scanners or scan parameters used and differences in subject populations [6]. Recently, several guides have suggested using data gathered from multiple institutions to develop medical image processing based on artificial intelligence [7, 8]. Several research groups, including our group, clarified that training using data collected from multiple institutions can improve the generalization of CADe software to external datasets [9–11].

**Fig. 1:**
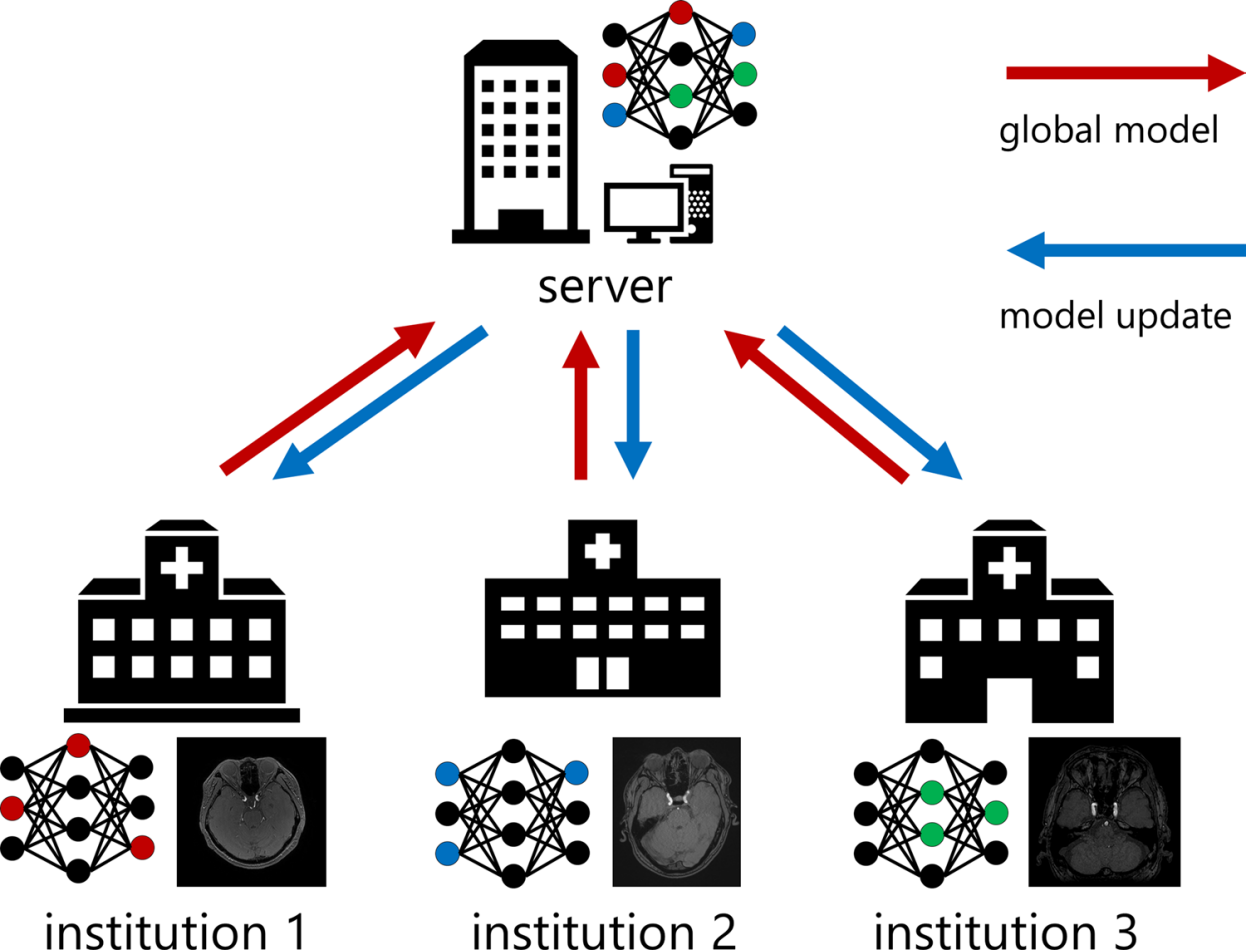
Structure of federated learning (FL)

**Fig. 2:**
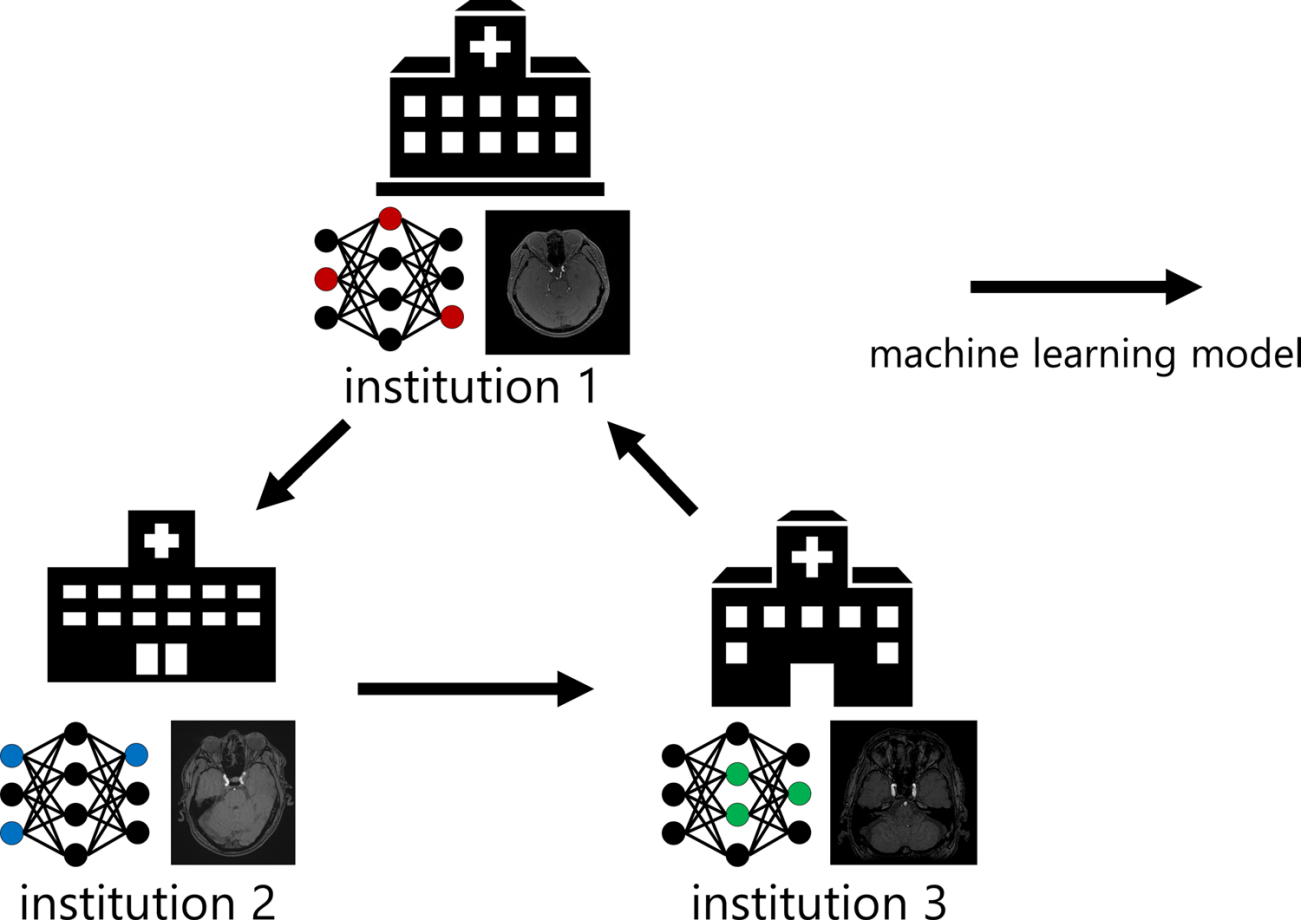
Structure of cyclical weight transfer (CWT)

Current CADe software development strategies typically employ a centralized strategy (hereafter, Centralized), in which the development is conducted at a development institution after the collection of de-identified data from multiple institutions [12]. In recent years, distributed learning, which involves sharing a machine learning model among institutions while keeping data locally, has attracted attention. This includes federated learning (FL) [13] (Fig. 1) and cyclical weight transfer (CWT) [14] (Fig. 2). In FL, a single machine learning model is trained simultaneously at all institutions and combines the model updates on a server to generate a global model that reflects the data of each institution. FL was designed to address situations such as (1) uneven numbers of cases among different facilities and (2) a large number of institutions, but with no guarantee that all will participate. However, FL demands a trustworthy server. FL is used in medical image analysis; for instance, in the classification of skin diseases in color images [15], classification of breast cancer in mammography images [16], and segmentation of head and neck cancer in ^18^F-fluorodeoxyglucose positron emission tomography/computed tomography images [17]. In contrast, in CWT, after training a model for a predetermined number of epochs at one institution, the model is sent to the next institution, for further training. This process is cyclically repeated among all institutions. CWT does not require a server and is suitable when the participating institutions are fixed. However, when there are variations in image quality between institutions, there tends to be instability in training, which can potentially lead to decreased performance of the model. Chang et al. evaluated CWT for image classification in these independent image collections (retinal fundus photos, mammography, and ImageNet) [14]. Although several research groups have reported the application of distributed learning to medical image processing tasks, to the best of our knowledge, there has been no report on the application of distributed learning to CADe software for head magnetic resonance (MR) images.

In this study, we investigated the feasibility of distributed learning for the development of lesion detection software for head MR images using multiple datasets with different scanner vendors and static magnetic field strengths. We targeted two types of CADe software for cerebral aneurysm detection in MRA images and brain metastasis detection in contrast-enhanced T1-weighted MR images.

## Materials and methods

We targeted two types of CADe software for cerebral aneurysm detection in MRA images and brain metastasis detection in contrast-enhanced T1-weighted MR images. Based on previous studies, we used different model architectures in each CADe software to validate the feasibility of distributed learning across multiple model architectures. Two distributed learning strategies (FL and CWT) were evaluated by comparison with the current CADe software development strategy (Centralized).

### Dataset

#### Cerebral aneurysm dataset

**Fig. 3:**
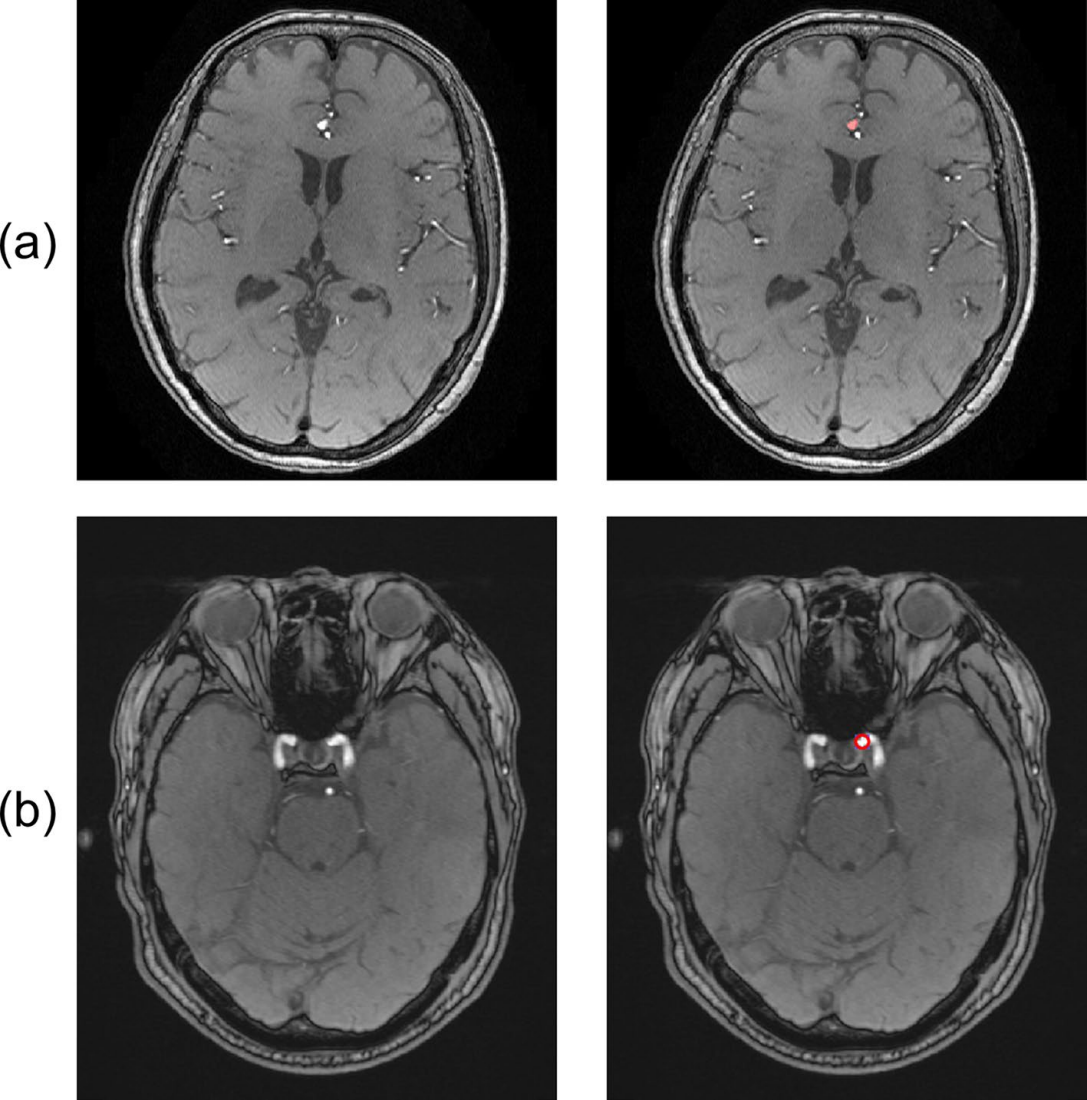
Example of cerebral aneurysms dataset. The original MRA images are shown on the left and the original MRA images with the gold standards are shown on the right. **a** 6 mm in anterior communicating artery (Dataset A, pixel-wise painting), **b** 5 mm in left internal carotid artery (Dataset B, spherical region)

This study was approved by the ethical review boards of our institutions. We collected a total of 315 MRA images from three in-hospital datasets. The details of the datasets are shown in Table 1. Figure 3 shows examples of cerebral aneurysm datasets. Each case included at least one aneurysm of 2 mm or more in diameter, which at least two experienced radiologists determined. For each aneurysm in Datasets A and C, one of two board-certified radiologists (N.H. and T.Y. with 32 and 26 years of experience in MRA interpretation, respectively) defined its area by pixel-wise painting. For each aneurysm in Dataset B, two board-certified radiologists (N.H. and S.M with 14 years of experience in MRA interpretation) defined its area via the spherical region of interests (ROIs) to encompass the entire aneurysms in 3D. Discrepancies between the two radiologists were resolved by a third board-certificated radiologist (T.Y.). These annotations were carried out using the web-based image database system CIRCUS DB [18]. Among the 105 cases for each dataset, 75 were used for training, whereas the remaining cases were equally assigned to validation and test sets. The details of the populations of the cerebral aneurysm dataset are shown in Table 2.

**Table 1:** Specifications of the cerebral aneurysm dataset

Dataset ID	No. of cases	MR scanners	Magnetic field strength [Tesla]	Scan parameters	Type of gold standard
A	105	two Signa HDxt and one Discovery MR750 (GE Healthcare, Waukesha, WI, USA)	3.0	Field of view (FOV), 240 mm; matrix size, 512 × 512 pixels; pixel spacing, 0.469 mm; slice thickness, 1.2 mm; slice interval, 0.6 mm; repetition time (TR), 22 or 25 ms; echo time (TE), 2.7–3.3 ms; flip angle (FA), 15°	pixel-wise painting
B	105	Skyra (Siemens Healthcare, Erlangen, Germany)	3.0	FOV, 240 mm; percent phase FOV, 82.3%; matrix size, 768 × 632 pixels; pixel spacing, 0.299 mm; slice thickness, 0.6 mm; slice interval, 0.6 mm; TR, 20 ms; TE, 3.69 ms; FA, 13°	spherical region
C	105	MRT200PP2 and Titan (Canon Medical Systems Co., Otawara, Japan)	1.5	FOV, 190 mm; matrix size, 512 × 512 pixels; pixel spacing, 0.371 mm; slice thickness, 1.0 mm for the MRT200PP2 and 0.5–0.6 mm for the Titan; slice interval, 0.5 mm for the MRT200PP2 and 0.5–0.55 mm for the Titan; TR, 28­–33 ms; TE, 6.8 ms; FA, 18°	pixel-wise painting

**Table 2:** Populations of the cerebral aneurysm dataset

Dataset	Dataset A			Dataset B			Dataset C		
	Training	Validation	Test	Training	Validation	Test	Training	Validation	Test
Demographics									
No. of patients	75	15	15	75	15	15	75	15	15
No. of MRA images	75	15	15	75	15	15	75	15	15
Age (year)	62 ± 11	60 ±11	61 ± 11	60 ± 12	59 ± 11	54 ± 14	61 ± 14	55 ± 16	58 ± 14
M/F ratio	36:39	8:7	10:5	42:33	9:6	12:3	34:41	10:5	7:8
Aneurysm information									
Multiplicity									
Single	61 (81.3)	11 (73.3)	13 (86.7)	67 (89.3)	14 (93.3)	15 (100.0)	65 (86.7)	15 (100.0)	14 (93.3)
Double	12 (16.0)	4 (26.7)	2 (13.3)	7 (9.3)	1 (6.7)	0 (0.0)	9 (12.0)	0 (0.0)	1 (6.7)
Triple	2 (2.7)	0 (0.0)	0 (0.0)	1 (1.3)	0 (0.0)	0 (0.0)	1 (1.3)	0 (0.0)	0 (0.0)
Location									
ACA territory	23 (25.3)	4 (21.1)	8 (47.1)	11 (13.1)	3 (18.8)	3 (20.0)	8 (9.3)	2 (13.3)	2 (12.5)
MCA territory	11 (12.1)	1 (5.3)	2 (11.8)	16 (19.0)	2 (12.5)	2 (13.3)	15 (17.4)	0 (0.0)	4 (25.0)
ICA territory	51 (56.0)	11 (57.9)	5 (29.4)	49 (58.3)	11 (68.8)	10 (66.7)	61 (70.9)	11 (73.3)	8 (50.0)
PCA territory	3 (3.3)	3 (15.8)	2 (11.8)	1 (1.2)	0 (0.0)	0 (0.0)	0 (0.0)	1 (6.7)	0 (0.0)
Others	3 (3.3)	0 (0.0)	0 (0.0)	7 (8.3)	0 (0.0)	0 (0.0)	2 (2.3)	1 (6.7)	2 (12.5)
Diameter [mm]^b^									
2	35 (38.5)	6 (31.6)	6 (35.3)	18 (21.4)	2 (12.5)	1 (6.7)	71 (82.6)	11 (73.3)	8 (50.0)
3	22 (24.2)	7 (36.8)	7 (41.2)	39 (46.4)	6 (37.5)	6 (40.0)	8 (9.3)	3 (20.0)	3 (18.8)
4	16 (17.6)	5 (26.3)	3 (17.6)	17 (20.2)	3 (18.8)	7 (46.7)	2 (2.3)	1 (6.7)	0 (0.0)
≥5	18 (19.8)	1 (5.3)	1 (5.9)	10 (11.9)	5 (31.3)	1 (6.7)	5 (5.8)	0 (0.0)	5 (31.3)

Continuous variables are shown as the mean ± standard deviation. Categorical variables are shown as the number and percentage (in parentheses)

*MRA* magnetic resonance angiography, *ACA* anterior cerebral artery, *MCA* middle cerebral artery, *ICA* internal carotid artery, *PCA* posterior cerebral artery

#### Brain metastasis dataset

**Fig. 4:**
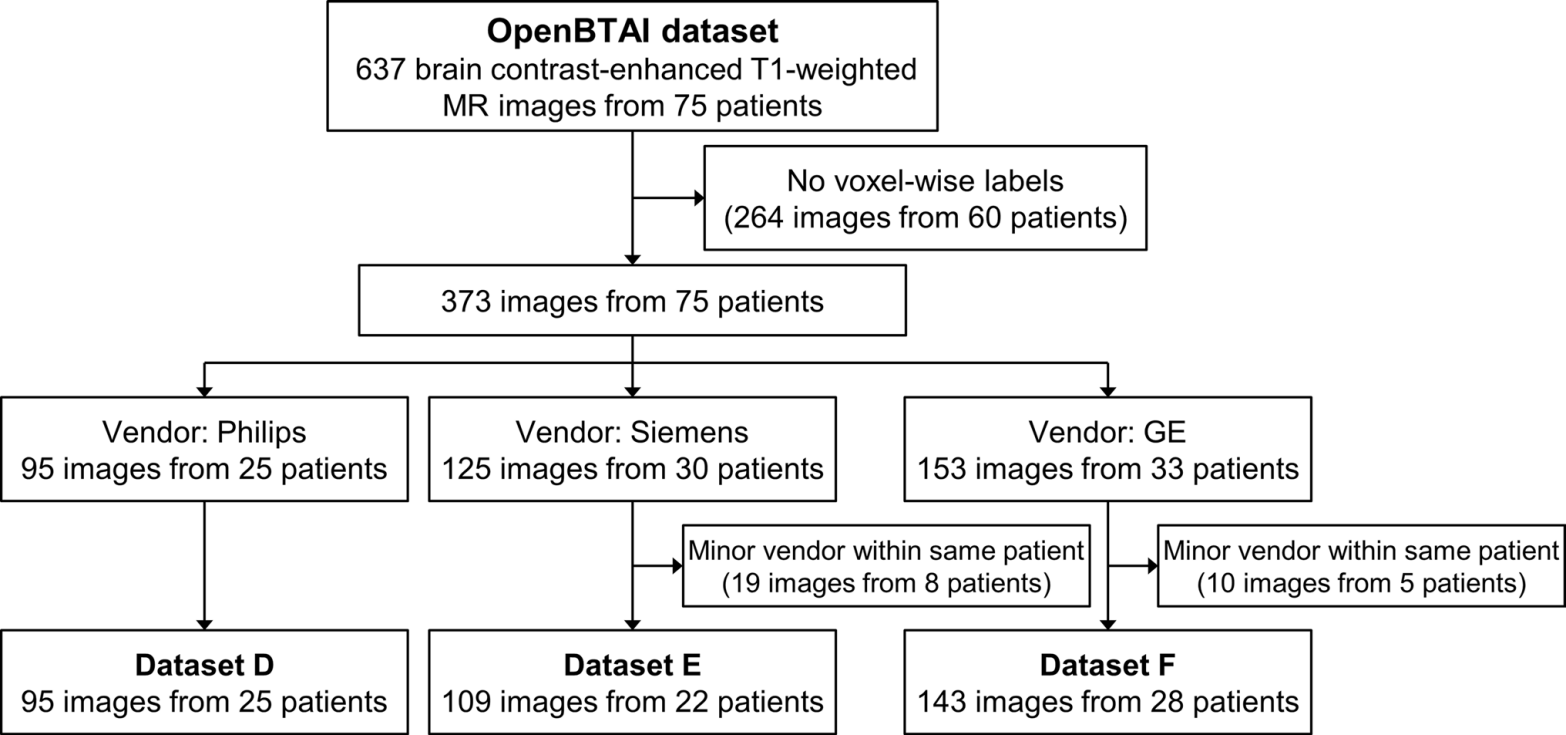
Flowchart of image selection for the brain metastases dataset

**Fig. 5:**
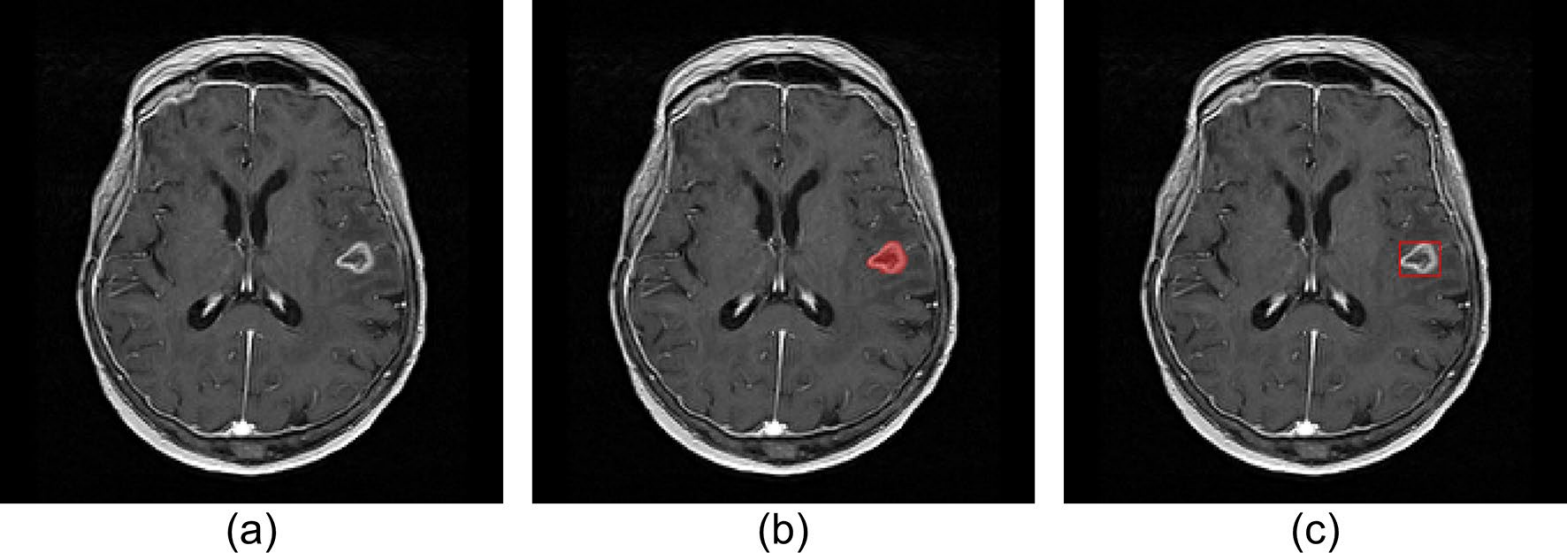
Example of brain metastasis dataset. **a** Original contrast-enhanced MR image (metastasis of 20.5 mm in diameter), **b** voxel-wise labeled image (red region) overlaid on the original MR image, and **c** bounding box (red rectangle) defined from voxel-wise labeled image

We selected 347 brain contrast-enhanced T1-weighted MR images from 75 patients in the open database of brain tumors for studies in artificial intelligence (OpenBTAI) brain metastasis dataset [19] that satisfied two conditions: (1) manually defined voxel-wise labels of metastasis stored in The Neuroimaging Informatics Technology Initiative (NIfTI) format are available at the Figshare Repository[20], and (2) in patients whose images were acquired using scanners from multiple vendors, only the images from the vendor with the most number of series are used (Fig. 4). The details of the datasets are presented in Tables 3 and 4. Figure 5 shows an example of the brain metastasis dataset.

**Table 3:** Specifications of the brain metastasis dataset

Dataset ID	No. of cases	MR scanners	Magnetic field strength [Tesla]	Scan parameters
D	20	Panorama (Philips Healthcare, Best, The Netherlands)	1.0	Field of view (FOV), 224 or 240 mm; matrix size, 512 × 512 or 560 × 560 pixels; pixel spacing, 0.429 or 0.438 mm; slice thickness, 1.6 mm; slice interval, 0.8 mm; repetition time (TR), 25 ms; echo time (TE), 6.9 ms; flip angle (FA), 30°
		Intera and Ingenia (Philips Healthcare)	1.5	FOV, 230–280 mm; matrix size, 256 × 256–640 × 640 pixels; pixel spacing, 0.406–0.977 mm; slice thickness, 1.0–4.0 mm; slice interval, 0.8–2.0 mm; TR, 25 ms; TE, 3.8–6.8 ms; FA, 30°
		Achieva (Philips Healthcare)	3.0	FOV, 230–250 mm; matrix size, 400 × 400–448 × 448 pixels; pixel spacing, 0.429 or 0.438 mm; slice thickness, 1.0 mm; slice interval, 1.0 mm; TR, 5.7­–5.8 ms; TE, 2.6–2.7 ms; flip angle (FA), 8°
E	22	Symphony (Siemens Healthcare)	1.5	FOV, 230–250 mm; matrix size, 400 × 400 pixels; pixel spacing, 0.5 mm; slice thickness, 1.3 mm; slice interval, 1.0 mm; repetition time, 11 ms; echo time, 4.8 ms; FA, 25°
F	27	Optima MR450w, Signa Artist, Signa EXCITE, Signa Explorer (GE Healthcare)	1.5	FOV, 200–280 mm; matrix size, 256 × 256 or 512 × 512 pixels; pixel spacing, 0.391–0.977 mm; slice thickness, 1.0–1.5 mm; slice interval, 0.5–2.0 mm; TR, 7.9­–600 ms; TE, 2.9­–16.7 ms; FA, 12–90°
		Signa EXCITE (GE Healthcare)	3.0	FOV, 260 mm; matrix size, 512 × 512 pixels; pixel spacing, 0.508 mm; slice thickness, 1.0 or 1.8 mm; slice interval, 0.5 or 0.9 mm; TR, 7.81­–8.83 ms; TE, 3.2 or 3.8 ms; FA, 10°

**Table 4:** Populations of the brain metastases dataset

Dataset	Dataset D			Dataset E			Dataset F		
	Training	Validation	Test	Training	Validation	Test	Training	Validation	Test
Demographics									
No. of patients	15	5	5	13	4	5	18	4	6
No. of MR images	65	15	15	76	15	18	98	27	25
Age (year)	57 ± 10	61 ±3	62 ± 14	51 ± 9	55 ± 16	48 ±9	58 ± 10	64 ±9	63 ± 12
M/F ratio	4:11	1:4	3:2	7:6	0:4	2:3	8:10	1:3	2:4
Primary cancer types									
Lung	8 (53.3)	1 (20.0)	5 (100.0)	7 (53.8)	1 (25.0)	3 (60.0)	12 (66.7)	2 (50.0)	4 (66.7)
Breast	6 (40.0)	1 (20.0)	0 (0.0)	4 (30.8)	2 (50.0)	1 (20.0)	4 (22.2)	2 (50.0)	2 (33.3)
Melanoma	1 (6.7)	2 (40.0)	0 (0.0)	1 (7.7)	1 (25.0)	1 (20.0)	0 (0.0)	0 (0.0)	0 (0.0)
Others	0 (0.0)	1 (20.0)	0 (0.0)	1 (7.7)	0 (0.0)	0 (0.0)	2 (11.1)	0 (0.0)	0 (0.0)
Metastasis information									
Total no. of metastasis	135	27	33	91	26	34	149	27	35
Average no. of metastasis	2.1 ±2.0	1.8 ± 1.2	2.2 ±1.5	1.2 ± 0.5	1.7 ± 0.8	1.9 ±1.2	1.5 ±0.8	1.4 ±0.5	1.4 ± 0.5
Individual metastasis volume (cm^3^)	2.4 ± 4.3	2.7 ±4.8	1.9 ± 4.3	3.5 ±5.15	1.6 ±2.1	3.9 ± 6.3	4.1 ± 5.6	3.1 ±4.5	4.0 ± 6.5
Individual metastasis size (mm)	14.3 ± 10.0	16.6 ± 11.4	12.5 ± 8.5	18.7 ± 10.9	14.3 ± 8.5	20. ±7.5	18.6 ± 11.6	17.4 ±7.1	16.5 ± 11.1
< 3 mm	3 (2.2)	0 (0.0)	1 (3.0)	0 (0.0)	3 (11.5)	0 (0.0)	10 (6.7)	0 (0.0)	0 (0.0)
≤ 3 to < 10 mm	54 (40.0)	10 (37.0)	16 (48.5)	16 (17.6)	7 (26.9)	0 (0.0)	33 (22.1)	2 (7.4)	12 (34.3)
≥ 10 mm	78 (57.8)	17 (63.0)	16 (48.5)	75 (82.4)	16 (61.5)	34 (100.0)	106 (71.1)	25 (92.6)	23 (65.7)

Continuous variables are shown as the mean ± standard deviation. Categorical variables are shown as the number and percentage (in parentheses). Metastasis size is defined as maximum diameter of the largest region in the axial section

### CADe algorithm

#### Cerebral aneurysm detection in MRA images

**Fig. 6:**
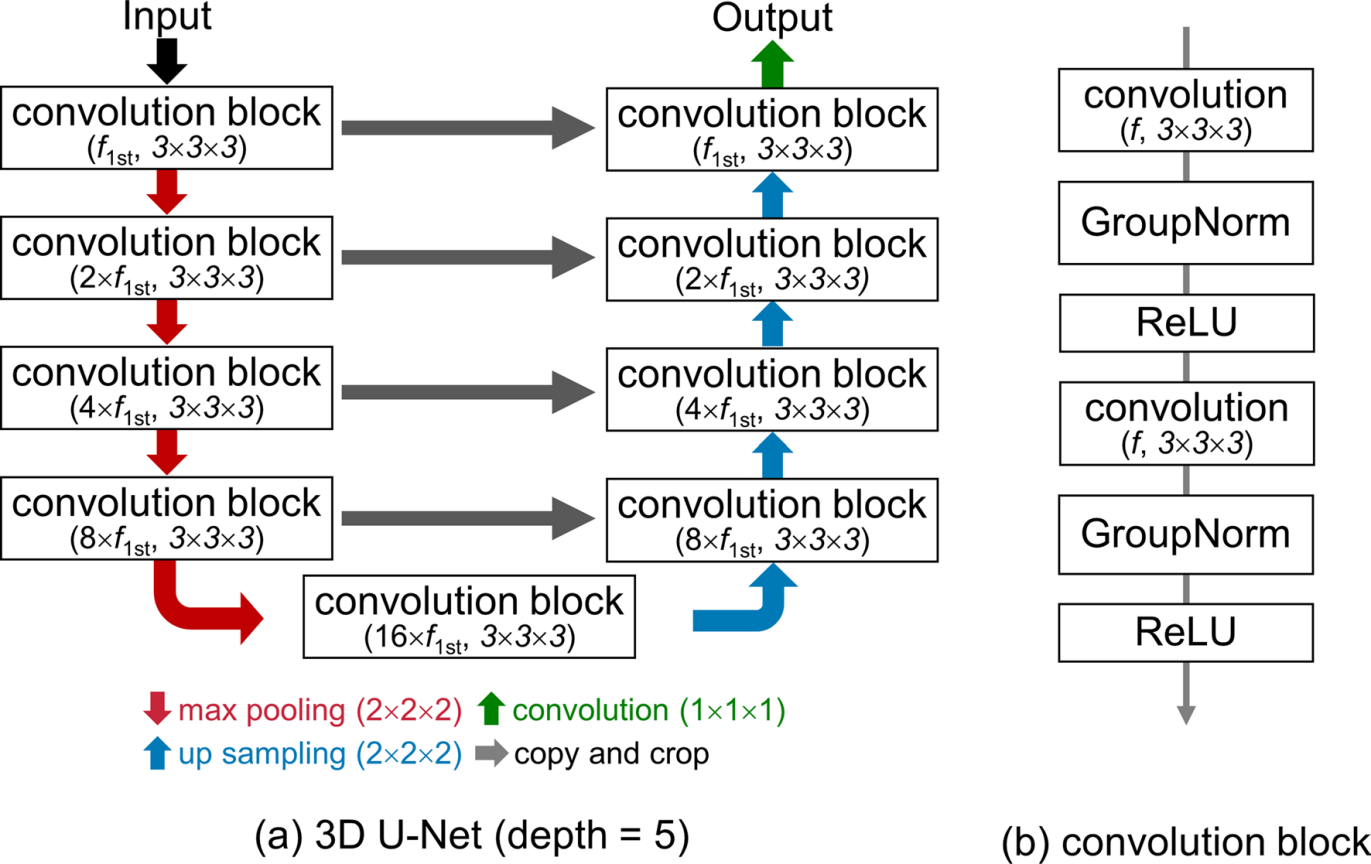
3D U-Net model used in cerebral aneurysm detection. **a** Overall step of 3D U-Net and **b** convolutional block: *f*_1st_, number of filters in the first layer; GroupNorm, group normalization with eight groups; ReLU, rectified linear unit

The input MRA images were first resampled to a 0.3 mm isotropic voxel size using tricubic interpolation, and the signal intensity distributions of images were standardized by the global piecewise linear mapping [21]. After that, we extracted arterial voxels from the MRA images by the region-growing-based method. We trained the 3D U-Net [22] model (Fig. 6) using each pair of the original MRA image and the area of aneurysms. We used the stochastic gradient descent (SGD) to optimize the network weights. We used group normalization [23] with eight groups in the convolution block. For each aneurysm, a 48 × 48 × 48 cubic volume of interest (VOI) around the center of gravity of the aneurysm region was extracted. In addition, four augmented VOIs are generated by random shifts within ± 24 voxels on the *x*-, *y*-, and *z*-axes and random rotation (0°/90°/180°/270°) in each of the axial, coronal, and sagittal planes. The augmented VOIs were changed for each epoch. In the detection phase, 48 × 48 × 48 cubic VOIs were extracted at lattice points (whose intervals are 24 voxels in each direction) in the bounding box of the extracted arterial volume, and all VOIs were fed into the trained 3D U-Net model. The outputs of the model are combined by taking the average of overlapped voxels. After that, the lesion candidates are extracted by binarizing the combined output and the following connected component analysis.

#### Brain metastasis in contrast-enhanced MR images

**Fig. 7:**
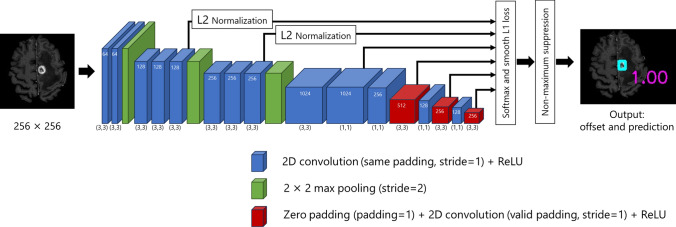
SSD model used in brain metastasis detection. *ReLU* rectified linear unit

Brain contrast-enhanced T1-weighted MR images were preprocessed using the following pipeline: (a) skull stripping employing the FSL Brain Extraction Tool (BET) ver.6.0.6.4 (FMRIB Centre, Oxford University, Oxford, UK) [24], (b) Gaussian intensity normalization [25, 26], wherein voxel values were divided by the standard deviation of the voxel values for each image, (c) resampling to an isotropic voxel size of 1.0 mm using tricubic interpolation, and (d) padding or cropping the size of the axial section (*x-* and *y-*axes) to 256 × 256. We used the single-shot detector (SSD) [27]-based model proposed by Zhou et al. (Fig. 7) [28], which was trained to map axial MR slices to a set of bounding box predictions encompassing brain metastasis and associated detection confidences. We implemented the model by using pytorch-ssd [29]. The ground-truth bounding boxes for each slice of the training data were defined from the voxel-wise label data of the brain metastasis dataset, and the bounding boxes with a height or width of two voxels or less were excluded (Fig. 5c). Table 5 shows the number of axial slices used for training from each dataset. Each slice included at least one bounding box. In the training phase, we assigned a binary class label to each anchor or default box. The positive (brain metastasis) and negative (background) anchors were selected on the basis of the intersection over union (IoU) with the ground-truth bounding boxes. Anchors with an IoU of 0.2 or greater were labeled positive, whereas anchors with an IoU of less than 0.2 were labeled negative. We used the SGD to optimize the network weights. In addition, two augmented slices were generated by random expansion, random horizontal flip with a 50% probability, and random rotation (0°/90°/180°/270°). Other parameters for training the model were set in accordance with a study by Zhou et al. [28]. In the detection phase, the trained model was applied to each axial slice, and the output bounding boxes from adjacent outputs were stacked to construct a lesion candidate.

**Table 5:** Number of axial slices used for training of brain metastasis detection

Dataset ID	Training set	Validation set
D	1605	331
E	1514	325
F	2512	468
total	5631	1124

### Hyperparameter tuning of CADe software

We used a conventional strategy that trains the model using data collected from each institution (Centralized) as a baseline. The hyperparameter used for training each CADe software was determined by hyperparameter tuning in Centralized. The model of each lesion detection software was implemented using Python 3.8.10 and PyTorch 1.13.0 [30]. These processes were performed on a workstation consisting of an Intel Xeon Gold 6230 2.1 GHz 20-core processor with 384 GByte RAM, NVIDIA GeForce RTX8000 GPU, Ubuntu 20.04.5, NVIDIA Driver 530.41.03, CUDA 11.8.0, and cuDNN 8.7.0. We used a single GPU for the training of the models.

#### Cerebral aneurysm detection

We employed 50 trials of hyperparameter tuning with random search [31] and utilized the area under the curve (AUC) value of the free-response receiver operating characteristic (FROC) curve, with the upper limit of two false positives (FPs) per case, as an evaluation criterion. The tuned hyperparameters were the depth of 3D U-Net (3/4/5), the number of filters of the first convolution layer (*f*_1st_, 8–64, step: 8), the batch size (2–32, step: 2), and the learning rate of the SGD (10^−4^–10^−2^). The number of maximum epochs was set to 100. In the performance evaluation, if the lesion candidate met at least one of the following criteria, it was judged as a true positive (TP), which were the same criteria used in a study by Nomura et al. [11]. The judgments were processed automatically.

1. The distance between the center of gravity of the lesion candidate and the center of gravity or location of lesion center of any aneurysm was less than 3.0 mm.
2. The distance between the center of gravity of the lesion candidate and the center of gravity or location of lesion center of any aneurysm was less than the radius of the aneurysm.

#### Brain metastasis detection

We employed 30 trials of hyperparameter tuning with random search and utilized the AUC value of the FROC curve, with the upper limit of 10 FPs per case, as an evaluation criterion. The tuned hyperparameters were the batch size (4–64, step: 2), and the learning rate of the SGD (10^–4^–10^–2^). The number of maximum epochs was set to 100. In the performance evaluation, if the lesion candidate contains at least one voxel of the label of brain metastasis, it was judged as a TP, which was equivalent to the same criterion used in a study by Zhou et al. [28].

### Training of CADe software using distributed learning

We compared the following three training strategies for each CADe software:

- Centralized (baseline)
- FL [13]
- CWT [14].

In FL and CWT, parameters obtained through hyperparameter tuning in Centralized were used, excluding the parameters described later. To facilitate comparison between the FROC curves from different strategies in a single number, the competition performance metric (CPM) [32], which defines the average sensitivity at a predefined number of FPs/case (1/8, 1/4, 1/2, 1, 2, 4, and 8 FPs/case) along an FROC curve, was employed as the evaluation criterion. The bootstrap method [33] was applied to test the statistical significance of differences in CPM between distributed learning and Centralized. The images were sampled 10,000 times, with each bootstrap containing the same number of images as in the original set. At each bootstrap iteration, the FROC curves were recalculated for each training strategy, and the ΔCPM was evaluated between each distributed learning and Centralized. *P* values were calculated as the fraction of evaluated metric populations that were negative or zero, corresponding to cases where the distributed learning did not outperform Centralized under comparison (null hypothesis). Differences in performance were considered statistically significant at *p* < 0.05. Statistical analyses were conducted using Python 3.8.10, along with the NumPy (version 1.25.2) and SciPy (version 1.11.1) libraries.

#### Federated learning (FL)

The framework of FL is a server-institution architecture, which consists of one server and *k* institutions, as shown in Fig. 1. In FL, a global model is sent from the server to each institution to train using its local data. The server combines model updates to generate a new global model that is sent back to the local institution for further training. This flow is defined as one round. We used the federated averaging (FedAvg) [13] generalized approach to update the global model. The number of epochs per round for FL was set to *E*_*FL*_ = {1/2/4}, and the maximum number of rounds was set to ⌈100/*E*_*FL*_⌉ rounds. Here, ⌈*x*⌉ denotes the smallest integer greater than or equal to *x*.

#### Cyclical weight transfer (CWT)

In the CWT framework, as shown in Fig. 2, there is no server and each institution conducts training using its own data. First, the order of institutions for training was determined and an initial model was provided for the first institution. In each institution, the model received from the previous institution is trained using its own data, and the trained model is then sent to the next institution. This process is cyclically repeated among all institutions until the termination criteria are met. The number of epochs per institution for CWT was set to *E*_*CWT*_ = {1/2/4}, and the maximum number of epochs was set to 100.

## Results

**Fig. 8:**
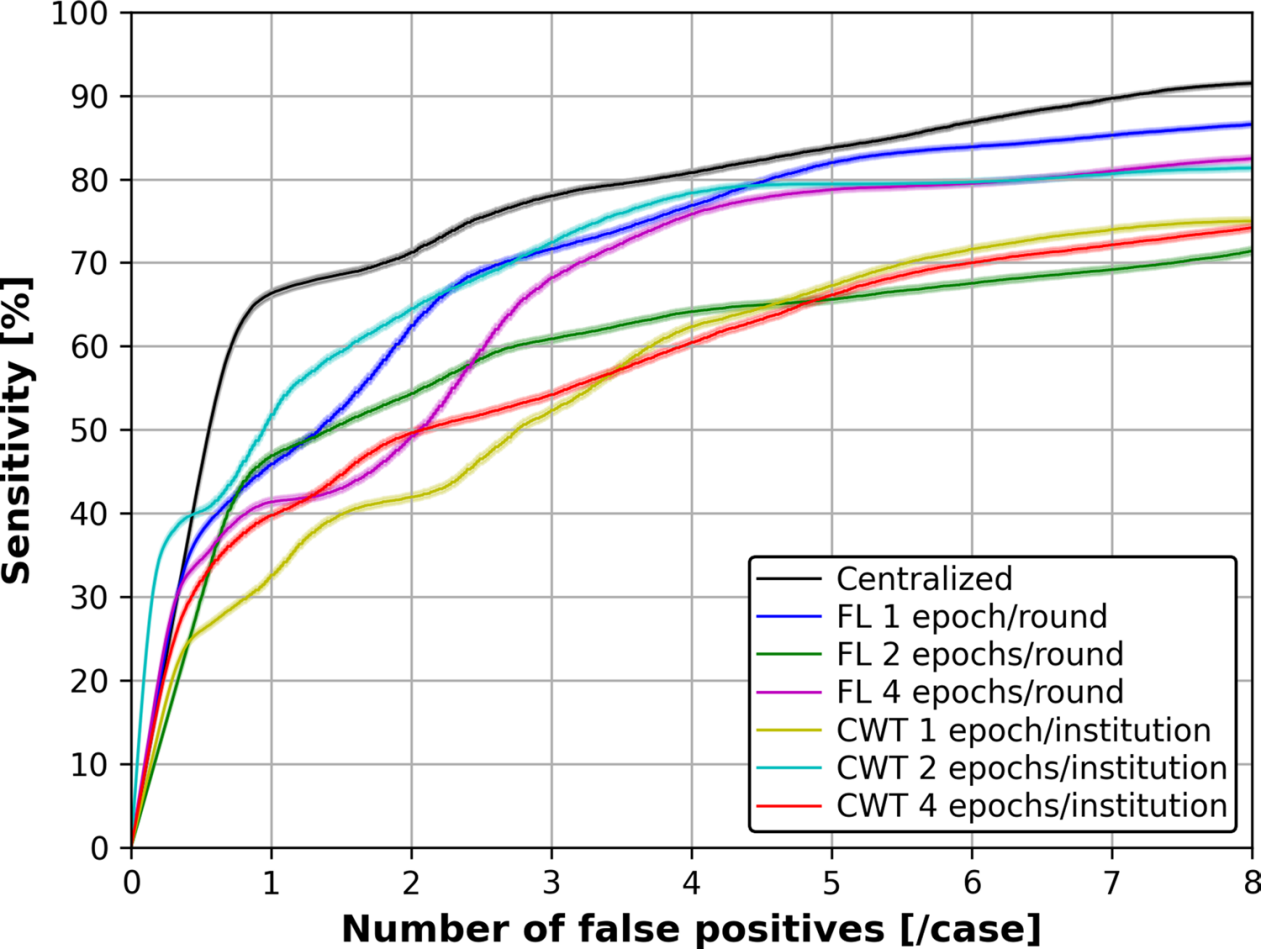
Average FROC curves obtained from 1000 bootstrap iterations for each training strategy in cerebral aneurysm detection. Confidence bands (semi-transparent) indicate 95% confidence intervals along the sensitivity axis

Figure 8 shows the FROC curves for each training strategy in cerebral aneurysm detection, and Table 6 shows the CPM scores in cerebral aneurysm detection. From Table 6, the CPM scores were 0.462 ± 0.058 for Centralized, 0.434 ± 0.059 (*p* = 0.677) for FL of 1 epoch per round, and 0.506 ± 0.061 (*p* = 0.200) for CWT of 2 epochs per institution. However, there was no significant difference in the bootstrap method results.

**Table 6:** CPM score for each training strategy in cerebral aneurysm detection

Training strategy		CPM score (mean ± standard deviation)	*p*-value
Centralized		0.462 ± 0.058	
FL	1 epoch/round	0.434 ± 0.059	0.677
	2 epochs/round	0.352 ± 0.055	0.985
	4 epochs/round	0.410 ± 0.056	0.909
CWT	1 epoch/institution	0.340 ± 0.049	0.993
	2 epochs/institution	0.506 ± 0.061	0.200
	4 epochs/institution	0.379 ± 0.062	0.961

*FL* federated learning, *CWT* cyclical weight transfer

**Fig. 9:**
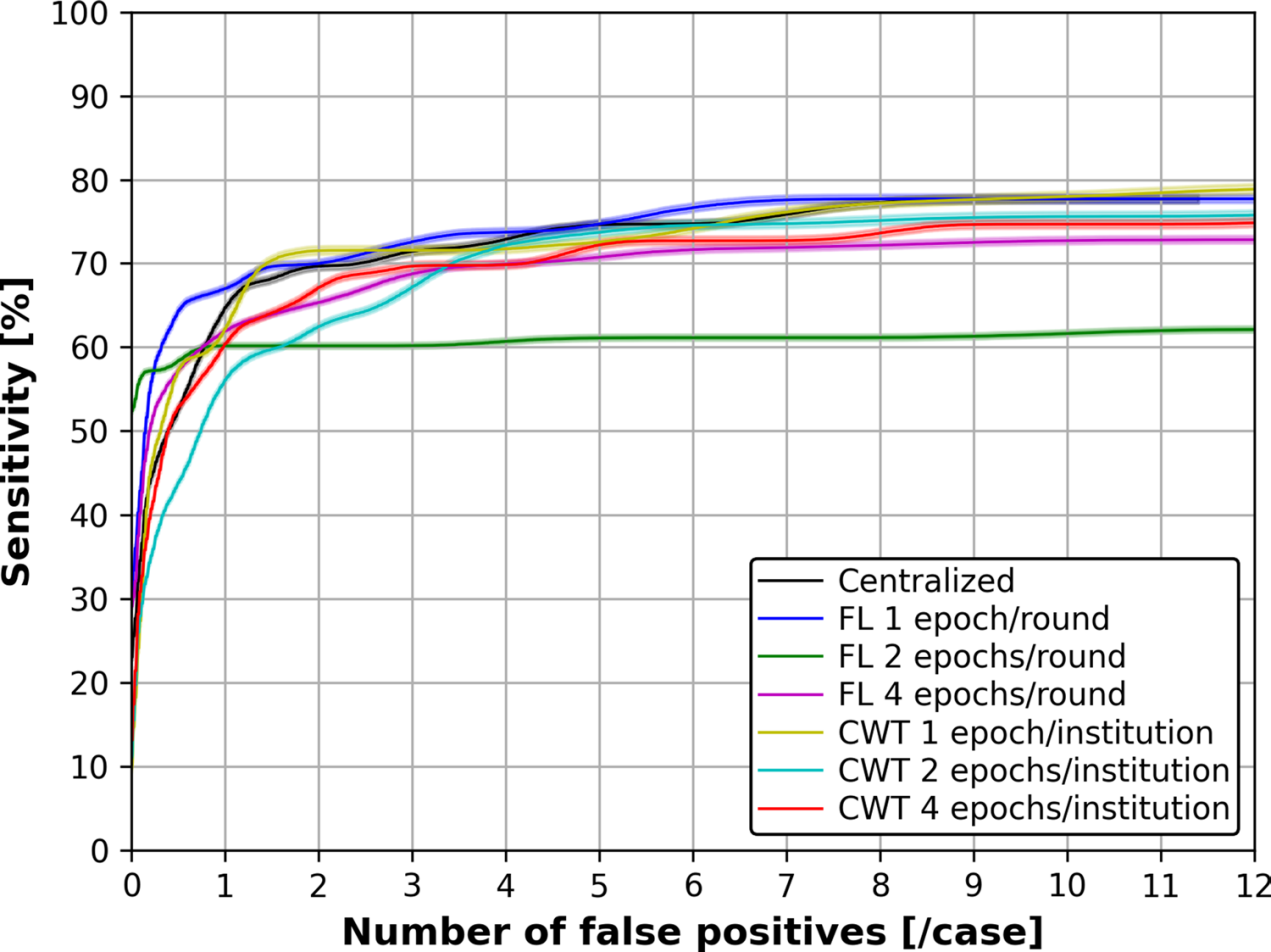
Average FROC curves obtained from 1000 bootstrap iterations for each training strategy in brain metastasis detection. Confidence bands (semi-transparent) indicate 95% confidence intervals along the sensitivity axis

Figure 9 shows the FROC curves for each training strategy in brain metastasis detection, and Table 7 shows the CPM scores in brain metastasis detection. From Table 7, the CPM scores were 0.602 ± 0.064 for Centralized, 0.654 ± 0.063 (*p* = 0.098) for FL of 1 epoch per round, and 0.603 ± 0.069 (*p* = 0.479) for CWT of 1 epoch per institution. However, there was no significant difference in the bootstrap method results.

**Table 7:** CPM score for each training strategy in brain metastasis detection

Training strategy		CPM score (mean ± standard deviation)	*p*-value
Centralized		0.602 ± 0.064	
FL	1 epoch/round	0.654 ± 0.063	0.098
	2 epochs/round	0.592 ± 0.051	0.582
	4 epochs/round	0.605 ± 0.050	0.450
CWT	1 epoch/institution	0.603 ± 0.069	0.479
	2 epochs/institution	0.538 ± 0.057	0.998
	4 epochs/institution	0.574 ± 0.061	0.926

*FL* federated learning, *CWT* cyclical weight transfer

## Discussion

We experimentally showed that the development of CADe software for head MR images utilizing data collected from multiple institutions, scanner vendors, and magnetic field strengths can be achieved through distributed learning. Unlike the centralized strategy, distributed learning does not require data sharing among institutions. In addition, the performance of CADe software trained through distributed learning was found to be equivalent to or better than that trained through the centralized strategy. If computational environments capable of training machine learning models, including deep learning, can be provided at each institution, distributed learning can become one of the strategies for CADe software development.

The performance of distributed learning depends on the number of epochs per training at each institution, that is, the epoch(s) per institution in CWT or the epoch(s) per round in FL. In CWT, the highest performance for cerebral aneurysm detection was achieved with two epochs per institution, and that for brain metastasis detection was achieved with one epoch per institution. These results are consistent with those of Chang et al. [14], which showed a tendency for better performance with fewer epochs per institution in their study. In FL, increasing the number of epochs for each round leads to an increase in loss in the latter stages of training [34]. In contrast, reducing the number of epochs per round increases the number of rounds required for loss convergence. Therefore, it is effective to decrease the number of epochs per round in the latter stages of training. Consequently, the number of epochs per training at each institution is one of the hyperparameters that should be adjusted in distributed learning. Recently, FL with hyperparameter optimization, which adjusts the learning rate and the number of epochs per round during the training, has been reported [34]. It is expected to improve the performance of CADe software when applying this method.

The distributed learning method that achieved the highest performance depends on the target CADe software. In CWT, updating the model parameters is performed only with data from the institution for training, which results in a tendency to depend on the last institution where training was performed. In contrast, FL is more stable than CWT, because FL updates the global model using updated information from all training institutions for each round [35]. The factors that showed different trends for each CADe software in this study include differences in machine learning tasks, models, or tendencies in data variations among institutions.

A common variability that is likely to arise in distributed learning is the difference in the amount of training data among institutions. For example, a setting in which both small (e.g., clinics) and large (e.g., university hospitals) institutions can be considered. In FL, FedAvg, a common approach for updating the global model, tends to reflect updates to the model weights from institutions with larger amounts of data. To address this issue, improved methods for updating the global model, such as FedProx [36] and Scaffold [37], have been proposed. However, these methods require an appropriate setting of the optimization parameters and additional exchanges between the server and each institution. In CWT, the model is trained for a fixed number of epochs at each institution, which can lead to overfitting in institutions with less training data and underfitting in institutions with larger training data. Balachandar et al. reported that performance decreased owing to variations in the amount of training data across institutions in two types of tasks: diabetic retinopathy detection in retinal fundus photos and thoracic disease classification in chest radiographs [38]. They also demonstrated that adjusting the number of epochs at each institution according to the amount of training data effectively mitigated performance degradation. Consequently, it is necessary to investigate the variation in the amount of training data in each institution in the training of CADe software.

To maximize the performance of CADe software at each institution, it is valuable not only to have models that maximize generalization through distributed learning, but also to have models dedicated for each institution. The training strategies for models dedicated to each institution include training using the data only from its institution and the application of personalized FL [39]. Personalized federated learning collaboratively trains models specific to individual institutions, and several studies have shown its applicability to medical image analysis [40–43]. We plan to apply personalized FL to the training of CADe software.

In distributed learning, a machine learning model is shared between institutions instead of sharing training data, thereby protecting the privacy of the training data. However, model parameters exchanged among institutions conceal sensitive information that can be exploited in privacy attacks [44]. For example, model inversion attack [45–49] infers personal information, including image data, from the model parameters. Therefore, to comply with strict data protection regulations such as the General Data Protection Regulation (GDPR) in EU/UK [50], it is also necessary to protect models [44]. As a strategy to ensure the security of models in FL, the application of privacy-preserving techniques such as differential privacy [51, 52] and homomorphic encryption [53] has been proposed [54–56]. Differential privacy involves adding random noise to the model parameters in each institution to obscure certain sensitive attributes of the model before sending it to the server. Homomorphic encryption allows computations to be performed on encrypted data without needing a secret key to decrypt the ciphertext. In FL, each institution sends a model encrypted with homomorphic encryption to the server, and the server updates the global model using the encrypted models. We plan to investigate the application of these privacy-preserving techniques in the training of CADe software.

Our study has several limitations. First, we evaluated only two types of CADe software. The results may not be applicable to other lesions. Second, many hyperparameters used in distributed learning were obtained through hyperparameter tuning in Centralized. In particular, for brain aneurysm detection, the structure of the model is also included in the hyperparameters, and the optimal structure of the model may differ with distributed learning. Although FL combined with network structure search has also been proposed [57], further investigation is necessary to apply it to the model used in this study. Third, the amount of image data used with each CADe software was small. In machine learning, the impact of additional training data generally decreases as the amount of training data increases [58]. Pati et al. reported the training of a deep learning model for detecting glioblastoma sub-compartment boundaries using federated learning with 6314 multi-parametric MR images collected from 71 institutions across six continents [59]. In the federated setting, the results indicated that the performance improvement was not directly or linearly related to the amount of training data. Therefore, further investigation of the relationship between the amount of training data and the performance in distributed learning is required.

## Conclusion

We investigated the feasibility of distributed learning for the development of CADe software for head MR images. The results showed that distributed learning is feasible for training the model using data collected from multiple sites, scanner vendors, and magnetic field strengths. Our future works include validation using other types of CADe software, the application of personalized FL to maximize performance at each institution, and the introduction of privacy-preserving techniques such as differential privacy to enhance data security.

## Data Availability

Due to ethical restrictions imposed by the Research Ethics Committee of the Faculty of Medicine of the University of Tokyo, cerebral aneurysm dataset cannot be made publicly available. The original data of OpenBTAI dataset [19] are available at the Figshare Repository [20].
